# A Case of Eclampsia

**Published:** 1855-09

**Authors:** D. W. Stormout

**Affiliations:** Grandview, Ill.


### THE NORTH-WESTERN

### MEDICAL AND SURGICAL JOURNAL.

**NEW SERIES.**

VOL. IV. SEPTEMBER, 1855. NO. 9.

### ORIGINAL COMMUNICATIONS.

ARTICLE I.—*A Case of Eclampsia.* Read before the Esculapian Society, at the semi-annual meeting held in Marshal, May, 1855. By D. W. Stormout, of Grandview, Ill.

THE following case of Puerperal Convulsions is reported to the society, solely for the purpose of eliciting discussion as to the best treatment, in this somewhat rare, but very dangerous disease.

Was called to the country in the forenoon of April 9th, 1855, to visit Mrs. B. in labor with her first child; age 19, rather short, squarely built, short thick neck, florid complexion, dark hair and eyes. Ascertained the labor had set in about midnight. On examination, found the general condition of the patient normal; and the labor well advanced in the second stage, the head occupying the pelvic cavity. The pains, however, were irregular, and the case progressed slowly. She was delivered, between 12 and 1 o'clock, P. M., of a healthy male child, medium size. For more than an hour previously, while the head was engaged at the inferior strait and at the os externum, the patient was very restless, and complained much of the pains, which were, generally, not expulsive, but rather tetanic in their character There was no nausea, no perspiration about the face or thorax, and, in answer to the direct question, said she had no headache. After the birth of the child, she expressed herself as feeling quite easy, and conversed cheerfully. Traction at the cord showed the placenta still adherent. After waiting fifteen or twenty minutes—as is my custom frequently, where delay is not contra indicated,—and the pains not returning, I applied frictions over the fundus uteri. Violent contractions soon came on, and with them the extreme restlessness of the patient. She now complained, for the first time of severe headache, and was soon after thrown into violent convulsions. Ordered cold water to the head and face. Running the finger up the cord, I found the placenta engaged in the mouth of the womb, and hooking into it, extracted it immediately. The convulsions lasted about one minute. As soon as we could get things ready, I bound up the arm preparatory to bleeding. She was now lying in a semi-comatose condition, pulse slow, full, strong,—face flushed, purpleish,—breathing heavy. Had her raised partially erect, and succeeded in getting a full stream from a large orifice Bled to sickness and entire consciousness. Ordered cold water to be applied to the head aisiduously, sinapisms to the extremities. Gave calomel gr. xxv., Pulv. Rhei x. gr. One hour afterwards the headache returned severely. Had her raised to the erect posture, opened the vein, and again bled to sickness. Face pale—headache almost gone—feels better. Ordered a tablespoonfull of castor oil to be given in an hour, and repeated hourly until it operates.

Same day, 6 o'clock, P. M.—Has had six fits since I left—lies stupid; can be roused to consciousness for a moment only. Applied cups to back of neck and temples, but the fits returning, had to desist. Gave one drop of croton oil and repeated it at intervals until the bowels were freely moved twice about 10 o'clock ; dejections dark green. In the meantime, sent for Dr. Steele in consultation. The attacks continued to recur, and were increasing in frequency and intensity. The intervals more or less comatose ; face and eyes bloated ; patient unable to speak when aroused. Dr. S. now recommended forty drops of laudanum to be given every two or three hours, until the convulsions ceased, and continue cold water to the head and sinapisms to the extremities. I remained with the patient all night; and gave the laudanum every two hours. After taking the third dose, or about 4 o'clock, A.M., she passed from the stupor, in which she had been lying in the intervals, into a quiet sleep, which lasted about three hours. No return of the convulsions, of which she had twenty in fifteen hours; pupils dilated, countenance dull and heavy, skin moist, pulse 110, full, soft, compressible. Ordered the laudanum to be given if the fits returned or she became restless, and cold to the head unremittingly.

10th, 6 o'clock, P. M.—No return of the convulsions; has slept nearly all day; breathing natural; pulse 70; some fullness, but soft and compressible; skin moist; pupils not dilated; seems to be rational, but can not speak. The bowels not having been open since 11 o'clock last night, directed calomel and jalap, aa gr. x., to be repeated in three hours, and followed by castor oil, if necessary—Laudanum to be given pro re nata.

11th, 9 o'clock, A.M.—Rested well all night; medicine operated freely about midnight, dejections dark and offensive; pulse 70, soft; countenance still, somewhat dull; Converses some—head cool; but aches a little; feels sore all over: inclines to lie on the back with the knees drawn up. Tenderness on pressure over the uterus. Strong mercurial fector on the breath; disowns the child. ℞. Pulv. Doveri, gr. viii—every four hours—poultice of wheat bran and hops to the bowels, to be renewed every three hours.

12th, 10 o'clock, A. M.—Decidedly better. Has some appetite; countenance good; pulse 78; soft: skin moist; complains of the ptyalism; still some little tenderness over the the uturus on pressure, and some headache. Directed an astringent wash for the mouth, a dose of castor oil, and continue prescription as yesterday.

13th,—Still better. Milk coming; manifests some interest in the child to-day, for the first time.

She continued steadily to improve, and I discontinued my visit on the 15th.

Were there no premonitory symptoms of Eclampsia, in this case, which were unheeded; but which should have been met by appropriate treatment, and the attack thus warded off? On inquiring of Mrs. B., after convalescence, she informed me that her health had been good during gestation, with the exception of a headache, at times, during the last two months, which she ascribed to "cold." But this symptom was not present, until a very short time before the first convulsion, which was twenty minutes or more after the birth of the child. The bladder and rectum were both evacuated freely, just previous to my arrival. The labor was not more tedious than is frequently met with in primiparic, occupying but little over twelve hours. The uterine contractions, during the latter part of the second stage, were of rather a tetanic character, frequently causing loud complaints from the patient, with considerable jactation, and advancing the travail slowly. But this is not unusual with the muscular primiparous female. There was one symptom, however, which, taken in connection with the preceding, pointed to this attack with almost a certainty; that was the want of perspiration about the face and thorax But it was unaccompanied by any evidence of sanguine determination to the head, as cephalagia, vertigo, &c., and was thus unheeded. I have had two other cases recently, also in primiparous, muscular females, in which the uterine contractions were painful, irregular, and not expulsive, with jactation and no perspiration about the face and thorax, but accompanied, in both cases, with severe headache. A free bleeding promptly relieved all these symptoms in each case, and I verily believe, prevented an attack of Eclampsia.

There is much obscurity as to the cause of this disease. Perhaps we would cover the whole ground, if we would say the puerperal state is the predisposing, and sympathetic reaction upon the brain and nervous system from an irritation of the womb, is the exciting cause.

In the treatment there are some points upon which all are agreed, while upon others we find the strangest diversity of opinion; rendering it difficult often for the inexperienced practitioner to decide at once what course to pursue. For what he does, he must do promptly and decidedly, or he will loose his patient.

Nearly all insist in every case, upon free general bloodletting, with brisk purgatives, cold to the head, revulsives to the extremities, and cupping from the temples or back of the neck, where there is stupor. Here, if the convulsions continue, the paths diverge. One class, among whom Prof. Meigs stands pre-eminent, strongly insist upon the administration of full doses of opium. Others again, among whom are Dewees, Colombat, and Cazeaux, condemn this practice in most unqualified terms Cazeaux, in his excellent work on midwifery, &c., translated by Thomas,—page 612, says; "In my estimation, the opiates ought to be wholly banished from the treatment of a disease, which so often terminates in cerebral congestions." The learned author also tells us, page 608, "But these," (the encephalic congestion, extravasation or effusion, frequently observed *post mortem*,) "are evidently nothing more than secondary lesions, the effects, not the cause, of the convulsions." And again, ' often this disease leaves no anatomical lesion behind " Now, if the convulsions are the cause of death, and the lesion observed are the effects, not the cause of the convulsions, a rational treatment would adopt a remedy which would arrest this cause before these effects were produced, and thus remove the danger. Opium, in hypnotic doses, is that remedy. This might be inferred from the known therapeutical properties of this drug, to relieve spasms and quiet irritation. It is also confirmed by experience. But its administration should always be preceded by a free bleeding and brisk purging, with cold to the head, revulsives, &c.

Chloroform, by inhalation, is recommended in this disease. I would not hesitate, in another case, if the convulsions continued after V. S., to administer the anasthetic in sufficient quantities to arrest the paroxysms; or at least to check their force and frequency until they could be relieved by opium.

Such is an outline of the treatment I would adopt in this frightful disease, where the attack comes on after the completion of labor, or before its commencement. But if labor should set in, or the attack commence during its progress, in addition to this, I would use all proper means to expedite the delivery, and administer chloroform as freely as the safety of the patient would admit, or the frequency of the convulsions demanded.

May 15th, 1855.

